# Editorial for the Special Issue Entitled “Novel Aspects of Endocrine Care: From Patient-Centered Management of Endocrine Diseases to Guidelines Standards”

**DOI:** 10.3390/jcm15186993

**Published:** 2026-09-09

**Authors:** Mara Carsote, Claudiu Nistor, Ana-Maria Gheorghe

**Affiliations:** 1 PhD Doctoral School, “Carol Davila” University of Medicine and Pharmacy, 020021 Bucharest, Romania; 2 Department of Clinical Endocrinology V, “C.I. Parhon” National Institute of Endocrinology, 011863 Bucharest, Romania; 3 Department of Endocrinology, “Carol Davila” University of Medicine and Pharmacy, 020021 Bucharest, Romania; 4 Department 4-Cardio-Thoracic Pathology, Thoracic Surgery II Discipline, “Carol Davila” University of Medicine and Pharmacy, 050474 Bucharest, Romania; 5 Thoracic Surgery Department, “Dr. Carol Davila” Central Military University Emergency Hospital, 010242 Bucharest, Romania

**Keywords:** parathyroid, primary hyperparathyroidism, Turner syndrome, quality of life, fine needle aspiration tumor, endocrine, hormone

Endocrine care in the modern medical and surgical era is characterized in many societies by easy access to screening protocols (e.g., routine blood calcemic level testing), multi-modal imaging evaluation tools (including artificial intelligence-generated assessment data and algorithms) and minimally invasive surgical procedures (e.g., parathyroidectomy, thyroidectomy or adrenalectomy), which offer an overall optimal outcome in everyday cases according to current guidelines [1,2,3]. In addition to this standard approach, unusual/exceptional endocrine pathology (e.g., parathyromatosis, etc.) faces challenges associated with a low level of statistical evidence and tailored/individual multidisciplinary decision-making remains mandatory [4,5,6].

In this Special Issue, distinct aspects of endocrine management are addressed, such as the clinical and lab spectrum in primary hyperparathyroidism across real-life settings [7], quality-of-life exploration in relation to confirmed cases of Turner syndrome [8], the long-term impact of chronic hypoparathyroidism, specifically, its influence on renal function [9], and an exceptional case of disease resolution following a fine-needle biopsy in a parathyroid tumor [10].

Over the years, the phenotype in primary hyperparathyroidism has shifted to less severe forms, including asymptomatic or normocalcemic diseases, due to early diagnosis and management [11,12,13]. Nevertheless, osteoporosis and osteoporotic fractures, as well as kidney stones and nephrocalcinosis, are part of the conventional presentation in more than half of diagnosed adults [14,15,16]. Females are 2–3 times more often affected than males in non-genetic variants, while 1 out of 100 elderly seniors are potentially affected by the condition [17,18,19]. In addition to bone mineral density, bone quality is also impaired, as shown by a reduced trabecular bone score, with potential post-surgery improvement. In addition, the co-presence of type 2 diabetes, which is regarded as a non-conventional comorbidity, increases the risk of skeletal and muscle effects [20,21,22].

In this landscape, Grigorie D. et al. [7] introduced a large real-life analysis of more than 400 subjects diagnosed over two decades, confirming female predominance (88.6%), with the average age at first diagnosis being within the sixth decade of life. The reported rate of familial variants was 4.4%, which is less than the usual rate of 10%, and this might be connected to local protocols of genetic counseling [7,23]. Notably, the co-presence of a secondary component due to vitamin D deficiency remains a topic of interest, since the mean 25-hydroxyvitamin D was found in the deficiency ranges (of 17.95 ng/mL), while one out of five individuals with primary hyperparathyroidism showed synchronous severe hypovitaminosis D. Further, vitamin D supplementation protocols might impact these results [7,24]. Notably, old presentations of the parathyroid condition, such as osteitis fibrosis cystica/brown tumors, were reported in 1.7% of the cohort, which may be linked to under-diagnosis and delayed intervention in certain population sub-groups [7,25]. Additionally, another rather high rate was found for post-parathyroidectomy confirmation of a parathyroid carcinoma (of 5.2%), a topic that recently has been revised from the perspective of histological and immunohistochemistry criteria [7,26]. Interestingly, the authors provided an in-depth analysis of non-classical parameters and determined that more than half of the subjects presented cardiovascular anomalies, while glucose profile disturbances were identified in 12% of the study population. Overall, 11.38% of the patients presented a non-parathyroid malignancy, such as differentiated thyroid carcinoma or breast neoplasia, which raises the issue of incidental findings across performant imaging and pathological investigations [7,27].

Remarkably, exceptional reports of primary hyperparathyroidism remission have been associated with spontaneous parathyroid tumor evolution or associated with fine-needle aspiration after prior biological and imaging confirmation of the condition [28,29,30]. This is more commonly identified in cystic or mix (solid-cyst) parathyroid lesions and in those developing hemorrhage/necrosis. Whether the parathormone/calcium normalization is lifelong (as similarly described, for instance, in pituitary neoplasia-related apoplexy [31]) or transitory, it remains an open issue [32,33,34]. Under these circumstances, we mentioned the case reported by Wojciechowska-Durczynska K. et al. [10]. This was a 71-year-old woman confirmed to have primary hyperparathyroidism; this was determined via polydipsia and asthenia in addition to a complex cardiometabolic and osteoporotic panel. She underwent fine-needle aspiration biopsy of a nodule situated posterior to the left thyroid lobe, and, after an initial cytological report showing a thyroid origin, a repeat aspiration was performed with parathormone washout and confirmed the presence of a parathyroid tumor based on a very high level of >5000 pg/mL. While parathyroidectomy was recommended according to standard guideline criteria, pre-operatory localization with 99mTc sestamibi scintigraphy scan did not confirm any tracer uptake in association with the normalization of the mineral metabolism assays, which remained stable for another five months [10]. Parathyroid hormone washout is not standardized yet, and is not mandatory for routine pre-operatory investigations. However, current evidence suggests its usefulness in selected cases, requiring a highly skilled team [35,36,37].

Situated at the other end of the parathyroid spectrum, non-transitory hypoparathyroidism represents an orphan disease—an extremely challenging ailment with sub-optimal management based on using vitamin D and calcium replacements alongside (to a certain extent) parathormone-based drugs [38,39,40]. Regardless of whether it is genetic, autoimmune or surgical (following thyroidectomy or parathyroidectomy), the disruption of calcium phosphate homeostasis damages bone health, cardiovascular status and renal function and reduces quality of life [41,42,43]. Thus, determining the long-term consequences becomes essential. For example, López-Mezquita Torres E. [9] introduced a longitudinal study involving 100 individuals diagnosed with chronic hypoparathyroidism and showed that estimated glomerular filtration rate deterioration correlated with the disease duration. Comorbid urolithiasis and cardiometabolic conditions were additional contributors, but a disease duration of >15 years was associated with the development of chronic kidney disease (85.7% sensitivity and 71.9% specificity) [9].

The roadmap of endocrine disease-associated quality of life may extend to syndromic presentations, such as Turner syndrome, and novel care requires a standardization of quality-of-life assessment for patients’ benefit [44,45,46]. A recent meta-analysis from inception until July 2025 identified only twelve studies that addressed this genetic pathology versus control groups. The main factors that impacted quality of life were hormone replacement treatment, patients’ social support and the panel of comorbidities [46]. For instance, Krzyścin M. et al. [8] introduced a comparative study involving 30 patients with Turner syndrome versus 43 healthy controls, who presented statistically significantly lower scores on SF-36 questionnaire domains for several variables, such as general health (*p* < 0.001), vitality (*p* = 0.002), social functioning (*p* = 0.004), and mental health (*p* = 0.01), as well as a higher BDI-II score for depressive symptoms (*p* = 0.008). Among the panel of clinical factors, final height and hearing loss were the main determinants of these parameters [8].

In conclusion, future research across modern endocrinology will show the shift in the clinical phenotype in primary hyperparathyroidism toward mildly symptomatic disease with a lower burden, as expected due to prompt detection and interventions, while an active search for non-conventional complications might prevent their under-diagnosis and improve the lifelong prognosis. In addition, the novel wave of tools such as parathormone washout upon fine-needle aspiration will help to refine neck masses with indeterminate profiles during imaging exploration and, potentially, exceptional cases may benefit from a therapeutic solution via aspiration biopsy (but the configuration of direct contributors for such an outcome has yet to be fully understood). Emergent therapeutic solutions in permanent hypoparathyroidism are required to improve the prognosis of the disease, which currently displays less-than-ideal interventional strategies. Finally, standardized tools for quality-of-life evaluation in complex endocrine conditions (e.g., Turner syndrome) will reflect the merging of multidisciplinary care, the endocrine approach, as well as patients’ social insertion and adaptation.

## Data Availability

No new data were created or analyzed in this study. Data sharing is not applicable to this article.
